# An optical photothermal infrared investigation of lymph nodal metastases of oral squamous cell carcinoma

**DOI:** 10.1038/s41598-024-66977-z

**Published:** 2024-07-11

**Authors:** Safaa Al Jedani, Cassio Lima, Caroline I. Smith, Philip J. Gunning, Richard J. Shaw, Steve D. Barrett, Asterios Triantafyllou, Janet M. Risk, Royston Goodacre, Peter Weightman

**Affiliations:** 1 Department of Physics, Oliver Lodge Laboratory, University of Liverpool, Liverpool, L69 7ZE UK; 2 Department of Physics, University of Jeddah, Jeddah, Saudi Arabia; 3 Centre for Metabolomics Research, Department of Biochemistry, Cell and Systems Biology, Institute of Systems, Molecular and Integrative Biology, University of Liverpool, Liverpool, L69 7ZB UK; 4 Department of Molecular and Clinical Cancer Medicine, Liverpool Head and Neck Centre, University of Liverpool, Liverpool, L7 8TX UK; 5 Head and Neck Surgery, Liverpool University Foundation NHS Trust, Aintree Hospital, Liverpool, L9 7AL UK; 6 Department of Cellular Pathology, Liverpool Clinical Laboratories, University of Liverpool, Liverpool, L7 8YE UK

**Keywords:** Head and neck cancer, Biological physics

## Abstract

In this study, optical photothermal infrared (O-PTIR) spectroscopy combined with machine learning algorithms were used to evaluate 46 tissue cores of surgically resected cervical lymph nodes, some of which harboured oral squamous cell carcinoma nodal metastasis. The ratios obtained between O-PTIR chemical images at 1252 cm^−1^ and 1285 cm^−1^ were able to reveal morphological details from tissue samples that are comparable to the information achieved by a pathologist’s interpretation of optical microscopy of haematoxylin and eosin (H&E) stained samples. Additionally, when used as input data for a hybrid convolutional neural network (CNN) and random forest (RF) analyses, these yielded sensitivities, specificities and precision of 98.6 ± 0.3%, 92 ± 4% and 94 ± 5%, respectively, and an area under receiver operator characteristic (AUC) of 94 ± 2%. Our findings show the potential of O-PTIR technology as a tool to study cancer on tissue samples.

## Introduction

Significant progress has been made over several decades in the application of a wide range of vibrational spectroscopic techniques to the study of cancerous tissues^1,2^. These include Raman spectroscopy^2–7^, Fourier transform infrared (FTIR) spectral imaging^2,7,8^, attenuated total reflection (ATR) FTIR spectroscopy^9,10^, atomic force microscopy-infrared (AFM-IR) spectroscopy^9,11–13^, scattering scanning near-field optical microscopy (sSNOM)^14,15^ and aperture SNOM^16,17^. IR (and Raman) imaging techniques provide morphological information based on the intrinsic (bio-)chemical signatures of tissue samples without staining or labelling, whereas the traditional histological techniques (the gold standard for studying tissue morphology) rely on staining the tissue prior to expert human analysis. However, usually the spatial resolution of IR techniques is diffraction-limited at ~ 10 µm (at 1000 cm^−1^). While this limitation can be overcome by image fusion^18^ and near-field techniques^14–17^ the former is computationally intensive, and the latter is relatively slow.

Recently, a new technique, optical-photothermal IR (O-PTIR), has been developed which aims to improve the spatial resolution obtained in IR spectral images^19,20^. O-PTIR uses a pump-probe geometry, with the pump beam being a quantum cascade laser (QCL) device emitting in the mid-IR and the probe beam a continuous wave (CW) visible laser. The pump beam induces a thermal response within the sample, which is then probed by the probe beam. In this scheme, the spatial resolution is determined by the diffraction limit of the wavelength used as probe beam, which here is ~ 0.5 µm as a 532 nm laser was used as the probe beam. This improvement in the spatial resolution achieved with O-PTIR comes at the expense of a reduction in the speed of data acquisition compared to some other IR techniques. As result the technique is particularly well suited to high spatial and high spectral characterisation of cells^15,21–26^. It has been used to investigate the metabolism of bacteria^22,23,27^ and the metabolic heterogeneity of human cells^26^ making it possible to uncover the underlying mechanisms of metabolic related disease. O-PTIR studies have also provided insight into structural properties confirming the identification of *β*-sheet structures in Alzheimer disease neurons^15^ and, in combination with Raman spectroscopy, for composition analysis of mammary microcalcifications^28^. The technique can also be used to study sub-cellular structures in live cells under aqueous conditions^29–31^ and by exploiting the polarisation sensitivity of O-PTIR it has been shown to determine the orientation of collagen in bone marrow tissue^32^ and tendons^33^. O-PTIR has also been shown to distinguish between malignant and non-malignant lung cells deposited on the glass slides used in clinical practice and in this study O-PTIR spectra did not need to be corrected for the Mie scattering that often distorts FTIR spectra^21^.

Historically, FTIR microspectroscopy has been used extensively to study tissue samples, but imaging fine structures within these samples, such as individual cells, has been challenging due to poor spatial resolution. The enhanced spatial resolution of O-PTIR has resolved this problem by facilitating more detailed analysis of such structures, such as individual cells, in tissue samples but the reduced speed of the technique is a severe limitation in applying it to characterise larger tissue samples obtained from cancer biopsies. A notable exception is the recent study of tissue microarrays obtained from ovarian cancer biopsies by Gallela et al.^34,35^. They presented a robust study of samples from 78 patients suggesting that O-PTIR could develop into a label-free, quantitative method of classifying ovarian tissue sub-types.

In this work, we investigate and assess the potential of O-PTIR by applying it to samples of oral squamous cell carcinoma (OSCC) metastases in cervical lymph nodes, which have been well characterised using FTIR imaging and aperture SNOM^17^. In particular we demonstrate that the ratio of O-PTIR image intensities 1252 cm^−1^ and 1285 cm^−1^ is able to discriminate OSCC metastases from normal lymph nodes that are unaffected by metastasis with an accuracy comparable to that achieved with optical microscopy of haematoxylin and eosin (H&E) stained tissue (currently considered the gold standard for diagnosis). Previous FTIR studies coupled with a machine learning algorithm, Metric Analysis (MA)^17,36^, showed that this ratio of image intensities accurately discriminated between OSCC metastases and adjacent lymphoid nodal tissue with sensitivities, specificities and precisions of 98.8 ± 0.1%, 99.89 ± 0.01% and 99.78 ± 0.02%, respectively. It is important to note that the efficacy of 1252 cm^−1^ and 1285 cm^−1^ as discriminating wavenumbers is not dependent on the identification of the particular chemical moieties present in the tissues. On the basis of the compilation of Talari et al.^37^ the origin of these discriminating wavenumbers was attributed to DNA and collagen respectively^17^.

## Methods

### Tissues and ethical statement

Formalin-fixed, paraffin-embedded tissue blocks of OSCC cervical lymph node metastases were obtained from 46 patients who provided written informed consent. The experiments were conducted under the sponsorship of the University of Liverpool, and ethical approval was obtained from the Northwest, Liverpool Central Research Ethics Committee (REC number EC 47.01). All experiments were performed in accordance with the relevant guidelines and regulations.

### Sample preparation

Tissue microarrays (TMAs) were constructed using a Beecher MTA-1 tissue microarrayer with 1 mm diameter tissue cores taken from OSCC lymph node metastases and non-malignant tissue. The areas that were cored were selected using optical microscopy from routinely prepared histopathological sections. Adjacent sections of 5 µm thickness were cut from the TMAs and mounted onto calcium fluoride (CaF_2_) disks for O-PTIR imaging and onto charged glass slides for comparative histopathology. O-PTIR images were obtained after the sections had been deparaffinised using a series of xylene and absolute ethanol baths. Briefly, the discs were immersed in two baths of xylene for 10 min each, then subjected to three, 5 min absolute ethanol baths. Following this treatment, the samples were placed in a desiccator for 24 h. Haematoxylin and eosin (H&E) staining was performed on the section mounted on a glass slide from each TMA using standard protocols and digitised at 40 × magnification using a whole-slide scanner Aperio CS2 scanner (Leica Biosystems, Milton Keynes, UK).

### Optical photothermal infrared (O-PTIR) spectroscopy

IR measurements were acquired using an O-PTIR instrument in reflection mode in conjunction with a mIRage microscope, both from Photothermal Spectroscopy Cooperation (Santa Barbara, USA)^22^. The O-PTIR instrument employed a continuous 532 nm probe beam and a pulsed mid-IR source generated by a MIRcat-QT-2400 QCL with four modules (Daylight Solutions, San Diego, USA) as the IR pump beam. The QCL average power was varied between 1 and 25 mW for the spectral range 933–1802 cm^−1^ and 1972–2322 cm^−1^ at a repetition rate of 100 kHz. The probe beam, with a total output power of 200 mW, was aligned to be collinear to the mid-IR pump beam. To avoid photothermal damage, only 20% of the pump laser power and 24% of probe laser power were used in the experiments. The O-PTIR instrument was operated in a controlled environment where the temperature and humidity were regulated. The relative humidity was less than 1%. IR data were collected through a reflective Schwarzschild 40 × objective with a numerical aperture (NA) of 0.78 and images were obtained at a working distance of 8 mm. Point spectra were obtained in the reflection mode with a spectral resolution of 2 cm^−1^. Single-wavenumber images for wavenumbers 1252, 1285, 1540 and 1660 cm^−1^ were collected from whole TMA cores at 1 μm step sizes with scanning speeds of 1200 cm^−1^ s−^1^. The wavenumbers 1252 and 1285 cm^−1^ were selected based on their potential discrimination between OSCC and non-malignant tissue as revealed by the output of a machine learning algorithm^17^. The 1540 and 1660 cm^−1^ wavenumbers were selected as peaks attributed to Amide II and I, respectively. No further images were acquired at other wavenumbers due to long data acquisition times. Data collection parameters were controlled using the PTIR Studio software (Photothermal Spectroscopy Cooperation, Santa Barbara, USA).

### Data pre-processing

In the previous analysis of the diffraction-limited FTIR images, a machine learning algorithm (MLA) analysed the spectral differences between the OSCC metastasis and surrounding lymphoid tissue, but the spatial resolution did not equal that of H&E images^17^. The higher spatial resolution of the O-PTIR images enabled the analysis of both spectral and spatial information. Spectral data and single-wavenumber images were pre-processed as described below to eliminate irrelevant information arising from environmental factors, thermal effects, paraffin contamination, or variations in sample thickness. The pre-processing was carried out using the Python programming language.

In the spectral datasets, pixels that were affected by the high refractivity of the visible light laser or paraffin contamination were removed. This was followed by min–max normalisation. Subsequently, the Savitzky-Golay smoothing algorithm^38^ was applied using a kernel size of 11 points and a second-order polynomial degree followed by rubber-band baseline correction. Finally, the spectral data were mean-centred.

For single-wavenumber images, pixels with ‘spike’ intensities were replaced with the average intensity value from the neighbouring pixels. Principal Component Analysis (PCA) noise reduction was then applied using three principal components, and min–max normalisation of the images was performed with Amide I (1660 cm^−1^) as the reference. The contrast in single wavenumber images and 1252 cm^−1^/1258 cm^−1^ ratio images was enhanced using adaptive histogram equalisation implemented using the Scikit-image library^39^: the target image was divided into tiles; then, for each tile, a histogram of intensity values within that tile was computed. The histogram was then equalised within the tile, resulting in the stretching of the intensity values. Lastly, the equalised tiles were stitched back to form the enhanced image.

### Cluster analysis

PCA was implemented in Python using the Scikit-learn library^40^. The input feature data were created from ratio images that were saved as standard JPEGs. Three classes of labelled pixels were identified from regions on the adjacent H&E images by a consultant Oral Pathologist (AT) (OSCC lymph node metastasis, non-malignant lymphoid tissue and non-malignant nodal stroma tissue) and extracted from the images, resulting in a large dataset (~ 15 million data points in all 46 images). Feature data were transformed to PCA components, PC1 and PC2, and score plots were created to display PC1 *versus* PC2. PCA components were then used as input features to the K-mean clustering algorithm for the entire ratio image.

### Classification model and training details

A spatial-spectral classifier based on a hybrid convolutional neural network (CNN) and a random forest (RF) model was developed for classifying the 1252 cm^−1^/1285 cm^−1^ ratio images of the tissue^17^. For this analysis, ratio images of annotated regions of tissue were divided into 75 × 75 pixel ‘patches’, the smallest size compatible with the Inception V3 model^41^ used in this analysis. These patches were the basic input to the CNN-RF hybrid classifier, consisting of a CNN feature extractor and RF classifier. In the feature extractor part of the model, spatial features were extracted by fine-tuning the model architecture with ImageNet pre-trained weights. The model contains the following building layers: an input layer followed by convolutional layers, pooling layers and Inception modules that use multiple filters, allowing it to extract a wide variety of features from a single feature map. These blocks are followed by reduction modules, global average pooling layer, fully connected layers, dense layers and dropout layers. The classification part of the model, retraining the last layer of the fine-tuned model, was followed by a flattening layer to reshape the output tensor into a shape that can be forwarded into the RF classifier, which had 300 trees and weighted the features based on the Gini index split factor^40^.

Deep-learning model training requires a large dataset to result in effective feature learning, to minimise overfitting that leads to poor model generalisation on the test set, and to minimise an imbalance between this case–control study. The dataset size used in this study contained 3005 cancer image patches and 1002 non-malignant image patches, and to expand the dataset size, an augmented dataset was created using TensorFlow^42,43^ through patch flipping and rotating, which resulted in expanding the size of the dataset by five times. The image patches were split into three parts which ensured that each sample was distributed across all sets; the approximate proportion of 60% for the training set used to train the model, 20% for the validation set used for fine-tuning the model hypermeter during the training, and 20% for the test set that was unseen by the model used for providing model evaluation. The training and validation dataset was partitioned into 20 folds based on the unique sample identification numbers (patient IDs) by using the Group*K*Fold method from Scikit-learn^40^. This method ensured that each fold covered a distinct group of image patches where the model trains on *K*-1 folds and validates on the remaining one. Training the model based on the patient ID as a grouping factor allowed for training on diverse image patches (patient set), which may expand the model's performance and ability to generalise to unseen data. The test image patches that have never been involved in the model training (constituting 20% of the dataset) were used for final predictions and evaluation of the model performance in terms of accuracy, sensitivity, specificity, precision, F1, and area under the receiver operator characteristic curve (AUC-ROC).

## Results

### Comparison of O-PTIR images with H&E stained images

**Figure 1:**
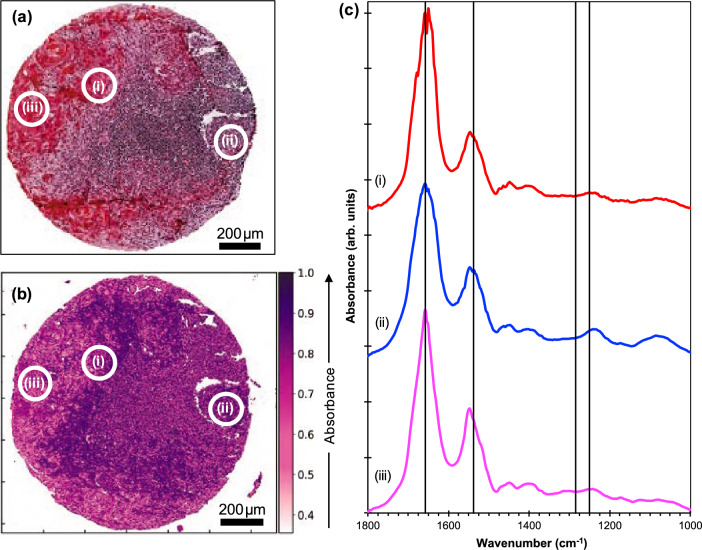
O-PTIR Spectra from points on a single TMA Core. (**a**) H&E image from an adjacent section for comparison; (**b**) O-PTIR image showing the averaged signal over four wavenumbers (1252 cm^−1^, 1285 cm^−1^, 1540 cm^−1^, and 1660 cm^−1^, indicated by vertical lines in (**c**)); and (**c**) O-PTIR spectra averaged over the areas of the circles shown in (**a**) and (**b**). (i) not heavily keratinised malignant tissue, (ii) lymphoid tissue and (iii) heavily keratinised malignant tissue.

Figure 1a is an optical image of a tissue core conventionally stained with H&E, which shows metastatic OSCC containing highly keratinised areas, together with lymphoid tissue. Figure 1b is an image of an adjacent slice of the same core obtained by combining and averaging the intensities of O-PTIR at each pixel for images obtained at 1252, 1285, 1540 cm^−1^, and 1660 cm^−1^ thereby generating a single averaged image for these vibrations from nucleic acids, collagens and Amide II and I, respectively^37^.

The O-PTIR instrument makes it possible to obtain complete IR spectra at specific points within an image. The three spectra shown in Fig. 1c were obtained from points selected from the H&E image (Fig. 1a). These correspond to (i) not heavily keratinised malignant tissue, (ii) lymphoid tissue and (iii) heavily keratinised malignant tissue. Within the range 1252 cm^-1^–1285 cm^−1^ there is a subtle difference between the average spectra of the three tissue types. In relation to the malignant tissue spectra, the lymphoid tissue spectrum showed a stronger peak at 1252 cm^−1^ and also a strong and distinctive band at 1085 cm^−1^. Both malignant spectra showed slight shifts to higher wavenumbers relative to the spectra of the lymphoid tissue in the Amide I band (7 cm^−1^) and in the Amide II band (18 cm^−1^). While these bands are broad, 1600–1700 cm^−1^ for Amide I and 1470–1600 cm^−1^ for Amide II, these shifts are significant.

**Figure 2:**
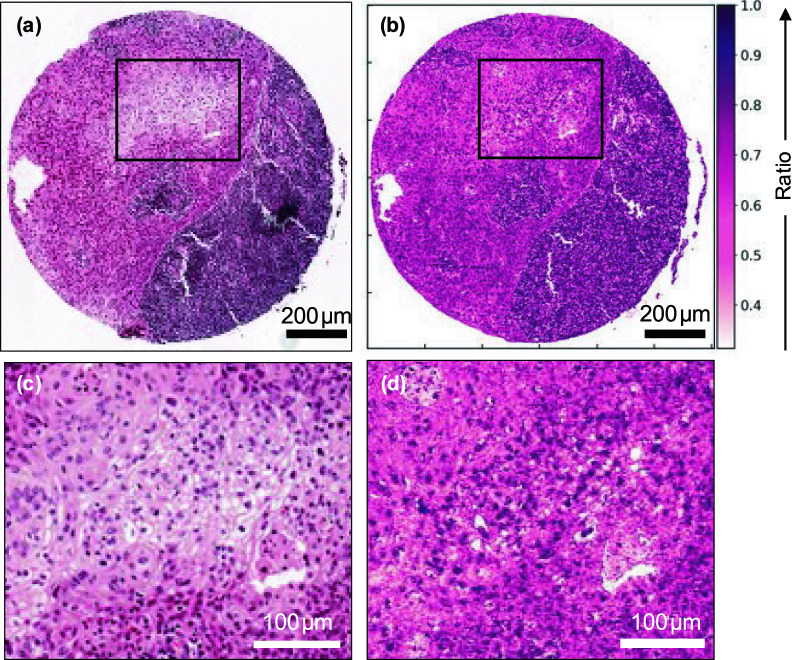
Comparison of resolution of H&E and O-PTIR ratio images. (**a**) H&E image (pixel size = 0.25 µm); (**b**) O-PTIR ratio image at 1252 cm^−1^/1285 cm^−1^ (pixel size = 1 µm); (**c**) Higher magnification of the rectangular area in (**a**); (**d**) Higher magnification of the rectangular area in (**b**). Lymphoid tissue appears as mostly dark purple and OSCC appears predominantly as pink. The colours used for the O-PTIR image were chosen to highlight the similarities with the H&E image.

Figure 2 shows a comparison of an H&E image (Fig. 2a) of a different core with the corresponding O-PTIR 1252 cm^−1^/1285 cm^−1^ ratio image (Fig. 2b). The ratio image shows excellent discrimination between the OSCC metastasis and lymphoid tissue, shown as pink and mostly dark purple respectively in Fig. 2a. Subsections of the images, denoted by the black rectangles, are shown at higher magnification in Figs. 2c and d. The comparison of the high-magnification images shows that the O-PTIR ratio images are able to reveal the cell nuclei.

**Figure 3:**
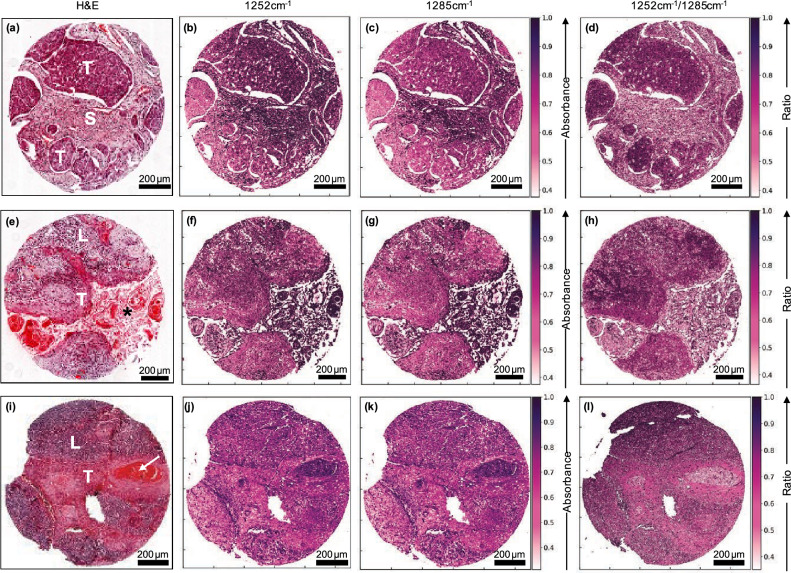
Discriminatory performance of the 1252 cm^−1^/1285 cm^−1^ ratio. Three separate lymph node metastases are shown (**a**–**d**, **e**–**h** and **i**–**l**). H&E images (pixel size = 0.25 µm) are shown in (**a**), (**e**) and (**i**); O-PTIR images at 1252 cm^−1^ (pixel size = 1 µm) are shown in (**b**), (**f**) and (**j**); O-PTIR images at 1285 cm^−1^ (pixel size = 1 µm) are shown in (**c**), (**g**) and (**k**); ratio of absorbance at 1252 cm^−1^/1285 cm^−1^ are shown in (**d**), (**h**) and (**l**). Key to tissue labels: lymphoid tissue (L); stroma (S); OSCC metastasis (T); heavily keratinised area (*); keratin pearl (arrow).

In order to explore the performance of the 1252 cm^−1^/1285 cm^−1^ ratio in discriminating metastatic OSCC from lymphoid tissue, a representative sample of the 46 cores was analysed further. The sample included three cores of varying histological features identified from H&E staining (Fig. 3). The first core, Fig. 3a, consisted of OSCC with no obvious keratin pearls, variously surrounded by stroma with little or no lymphoid tissue (approximately equal tumour/stroma ratio). The second core, Fig. 3e, consisted of OSCC with heavy keratinisation at the centre of tumour-cell aggregates and adjacent lymphoid tissue (increased tumour/lymphoid tissue ratio). The third core, Fig. 3i, consisted of OSCC with isolated keratin pearls, variously surrounded by lymphoid tissue (approximately equal tumour/lymphoid tissue ratio). The corresponding O-PTIR images obtained at 1252 cm^−1^ and at 1285 cm^−1^ are shown in Figs. 3b, c, f, g, j and k and the ratio images in Figs. 3d, h and l, respectively. The ratio images, shown in the fourth column of Fig. 3, show superior tissue discrimination to the single-wavenumber images and a similar level of tissue-type discrimination to that of the H&E stained images. Thus, when compared with the single-wavenumber images, the ratio images have a number of advantages: enhancing the difference between OSCC and surrounding stroma (Fig. 3d); allowing discrimination between lymphoid tissue and tumour rimming the heavily keratinised area (Fig. 3h) more similar to the corresponding H&E image; and emphasising the contrast between OSCC with keratin pearls and lymphoid tissue (Fig. 3l). In all three ratio images, the OSCC tumour can be distinguished from the surrounding tissues, confirming the efficacy of this ratio^17^ and the spatial resolution captured the tissue heterogeneity comparable with H&E images.

The improved spatial resolution of the O-PTIR images, ~ 0.5 µm, provides a better comparison with the H&E images than the diffraction-limited FTIR images, ~ 5–10 µm, obtained earlier^17^. Images formed from the ratio of intensities from two other combinations of single-wavenumber images, 1252 cm^−1^/1540 cm^−1^ and 1660 cm^−1^/1540 cm^−1^, showed some tissue discrimination, but these were inferior to that obtained with 1252 cm^−1^/1285 cm^−1^ (Supplemental Fig. S1). Ratio images formed from the combinations 1252 cm^−1^/1660 cm^−1^, 1285 cm^−1^/1540 cm^−1^, 1285 cm^-1^/1660 cm^-1^ showed little tissue discrimination (Supplemental Fig. S1).

### Cluster analysis

Further analysis of the H&E images of the 46 cores, of which those shown in Fig. 3 are only a representative sample, shows that while some cores are well described as mixtures of OSCC tumour and lymphoid tissue (Fig. 3i), others such as Fig. 3a have a more complex tissue structure containing stroma.

**Figure 4:**
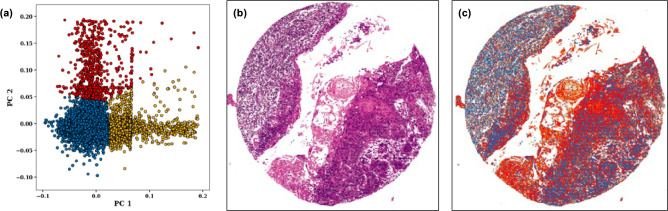
PCA score plot of three annotated classes. (**a**) Cluster plot of the 46 samples: OSCC metastasis (blue); lymphoid tissue (red); stroma (gold). (**b**) O-PTIR ratio image of a single core at 1252 cm^−1^/1285 cm^−1^. (**c**) O-PTIR cluster map corresponding to (**b**) showing tumour (red), cell nuclei (blue) and stroma (gold).

PCA of the annotated ratio images of the 46 cores resulted in a two-dimensional PC1 versus PC2 scatter plot of ratio pixel values that captured 79.7% of the total variance in the data set and revealed three main tissue types: OSCC metastasis, lymphoid tissue and stroma (Fig. 4).

### CNN-RF neural network analysis

The CNN-RF analysis captures the spectral and spatial information in the ratio images. The model was trained to discriminate between OSCC metastases and surrounding tissues with no discrimination between stroma and lymphoid tissue. The mean evaluation scores for the model are shown in Table 1 and suggest that the model is able to discriminate tumour from non-malignant tissue in this context.

**Table 1:** Mean evaluation scores for model discrimination performance between OSCC metastasis and non-malignant tissue.

Evaluation metrics	Mean score (%)
Accuracy	95 ± 5
Sensitivity	98.6 ± 0.3
Specificity	92 ± 4
Precision	94 ± 5
F1	97.7 ± 1.5
AUC-ROC	94 ± 2

Accuracy measures the proportion of correctly classified examples in both true positives and true negatives out of the total number of instances in the dataset. Precision measures the proportion of predicted positive examples that are actually positive. F1 combines precision and recall to provide a single value representing the balance between true positive and false positive rates in a classification task. The ± values are standard deviations.

## Discussion

In the present study, O-PTIR single-wavenumber images were collected at wavenumbers previously found to be important to discriminate OSCC nodal metastases from surrounding tissue based on results obtained in a previous work^17^ using FTIR spectroscopy (1252, 1285, 1540 and 1660 cm^−1^). The ratios of absorbances formed from the key discriminating wavenumbers found in the FTIR spectra, 1252 cm^−1^ and 1285 cm^−1^, are also the key ratios of absorbances in the O-PTIR spectra (Fig. 1). Due to the higher spatial resolution achieved by O-PTIR images, it was possible to capture morphological information from tissue samples that could not be assessed through traditional FTIR spectroscopy. These include the identification of cell nuclei (Fig. 2), which are important in the morphological study of cancers. Our findings also show that the ratio of O-PTIR images obtained at 1252 cm^−1^ and 1285 cm^−1^ were able to discriminate metastatic OSCC from other tissue types. A key contribution to this successful discrimination is the higher spatial resolution of the O-PTIR images, as compared to the original FTIR images.

Recently some insight into the chemical structure of these tissue types has been provided by the results of applying a regression-based fusion algorithm to merge an FTIR spectral image with an H&E image of OSCC metastasis in cervical lymphoid nodes^18,44^. This confirms that the success of the ratio of FTIR absorbance at 1252 cm^−1^ and 1285 cm^−1^ in discriminating between tissue types is due to the absorbance at these two wavenumbers being dominated by contributions from DNA and collagens, respectively, as originally suggested^17^. Furthermore, the expectation from histopathology and immunohistochemistry that cytokeratins should also make a significant contribution to OSCC was confirmed by a pixel-by-pixel fit of the fused spectral image to the FTIR spectra of collagens, DNA and cytokeratins^44^. This indicated varying contributions of these groups of molecules to the tissue types at high spatial resolution and, possibly, the subtle variation in contrast between Figs. 3d, h and l is influenced by those contributions.

Having established from this study of a larger cohort of samples that the variation in the contrast of O-PTIR 1252 cm^−1^/1285 cm^−1^ ratio images is dominated by contributions from at least three important groups of molecules, the question arises as to the likely success of this image ratio in broadly classifying tissues. The results of the CNN-RF analysis (Table 1), in which the O-PTIR images were classified in a binary system as metastatic OSCC or ‘other’ (which can loosely be regarded as a mix of lymphoid tissue and stroma) confirm the earlier analysis of FTIR spectral images^17^.

The PCA scores plots in Fig. 4 shows clusters corresponding to tissues that have similar or shared characteristics. The clusters are well separated, indicating clear differences in the chemical composition of the tissues. The overlap between clusters may be due to the sub-cellular components of cells within the tissues, such as cell nuclei. The comparison of Figs. 4b and c shows that the 1252 cm^−1^/1285 cm^−1^ ratio image shows the same tissue discrimination as that given by cluster analysis.

The results of our analysis are consistent with the earlier attribution of the 1252 cm^−1^ signal to the (PO_2_^−^) contribution from nucleic acids and the 1285 cm^−1^ signal to collagens^17,18,44^. Similar high-magnification images are available for all 46 cores examined and have potential to yield chemical insight into these tissue types and will be explored in future studies.

### Assessment of O-PTIR and comments on its use in a clinical setting

There is currently no suggestion for immediate application of O-PTIR in a clinical diagnostic setting. The data is presented as proof-of-principle only and extensive further validations would be needed before implementing O-PTIR in clinic.

The principal advantage of O-PTIR over other IR imaging techniques such as FTIR is that it improves the spatial resolution from ~ 5 − 10 µm to ~ 0.5 µm yielding chemical information on a sub-cellular length scale. This is comparable to the spatial resolution achieved in the visible region of the electromagnetic spectrum (400–700 nm) with H&E, ~ 0.25 µm, with a high-quality optical microscope. The synergy between IR spectroscopic and morphology information significantly improves the power of machine learning analysis. However, in order to be incorporated into clinical practice any new technique has to offer significant advantages over the long-established gold standard for clinical assessment of surgically resected tissues (i.e. conventional histopathology: H&E, special stains and immunohistochemistry). O-PTIR and related IR techniques are at an early stage of development and wavenumber combinations, such as those presented here have not yet been elucidated for other tumour types such as assessing the prognosis of oral epithelial dysplasia lesions^45,46^. It may also provide insight into the pattern of progression of a particular type of tumour in different anatomical sites and how this is influenced by physical and chemical variations in the microenvironment and/or etiological factors such as human papillomavirus (HPV) infection.

O-PTIR does not suffer from fluorescence, which can be a major problem of Raman spectroscopy, but it shares with Raman spectroscopy a dependence on spatially raster scanning the sample. This increases the signal acquisition rate for both techniques compared to the parallel detection techniques employed in state-of-the-art FTIR instruments. The increase in signal acquisition time of O-PTIR compared to FTIR is of the order of two orders of magnitude and as a consequence this usually limits the spectroscopic range of the analysis to a few carefully chosen wavenumbers. There have been recent advances in improving the speed of O-PTIR data acquisition. Reihanisaransari et al.^35^ have reduced the time taken to obtain O-PTIR images by an order of magnitude by combining an Amide I image obtained at high spatial resolution with infrared images (at selected wavenumbers) obtained at lower spatial resolution. By applying curvelet transforms, data fusion of the high- and low-frequency coefficients of the Amide I and selected images, respectively, allows the selected images to be reconstructed with relatively high fidelity. Yin et al.^31^ have increased the data acquisition speed over a small area by three orders of magnitude by synchronised raster scanning of the pump and probe lasers rather than raster scanning the sample. This made it possible to study dynamic changes in live samples.

To what extent O-PTIR can be applied in a clinical setting, particularly as a novel adjunct to standard histopathological techniques, remains to be seen. The stain-free component would be desirable as it would simplify the workflow, but the adoption of this technique would be influenced by validation, logistics and time/infrastructure needed for the O-PTIR analysis.

## Conclusion

An earlier study restricted to metastatic OSCC and lymphoid tissue showed that the ratio of absorbances obtained at 1252 cm^−1^ and 1285 cm^−1^ in FTIR images enabled discrimination between these two tissue types with high sensitivity and specificity^17^. The present investigation of a larger cohort of samples revealed, via O-PTIR, that the tissue cores include significant contributions from three tissue types: tumour, lymphoid tissue and stroma. The investigation also showed that O-PTIR 1252 cm^−1^/1285 cm^−1^ ratio images reveal subtle variations in image contrast arising from different combinations of three main tissue types, similar to those that are perceived in H&E images. The improved discrimination of the O-PTIR ratio images over the corresponding FTIR ratio images arises from the superior spatial resolution of the O-PTIR technique. The fine detail revealed in these images have potential to yield insight into the chemical composition of these tissue types. This will be pursued in future studies using this spatially sensitive technique.

The combination of O-PTIR images with a hybrid convolutional neural network and random forest analysis of the 1252 cm^−1^/1285 cm^-1^ ratio images yielded sensitivities, specificities, precision and an AUC-ROC of 98.6 ± 0.3%, 92 ± 4%, 94 ± 5% and 94 ± 2%, respectively. The present investigation has highlighted the potential of O-PTIR as a stain-free tool for tissue identification in OSCC nodal metastasis. It represents an innovative advancement as the first label-free approach to translate knowledge from FTIR into O-PTIR for classifying tissue types in OSCC nodal metastases.

### Supplementary Information

Supplementary Figure S1.

## Data Availability

The datasets generated during and/or analysed during the current study are available from the corresponding author on reasonable request.

## Code availability

The computer code is available from the lead author on reasonable request.
