# Supplementary material for: An optical photothermal infrared investigation of lymph nodal metastases of oral squamous cell carcinoma
Source: Sci Rep. 2024 Jul 11;14:16050. doi: 10.1038/s41598-024-66977-z (PMC11239877; doi:10.1038/s41598-024-66977-z)
Supplement: Supplementary file 1 — Supplementary Figure S1. [file 41598_2024_66977_MOESM1_ESM.pdf]

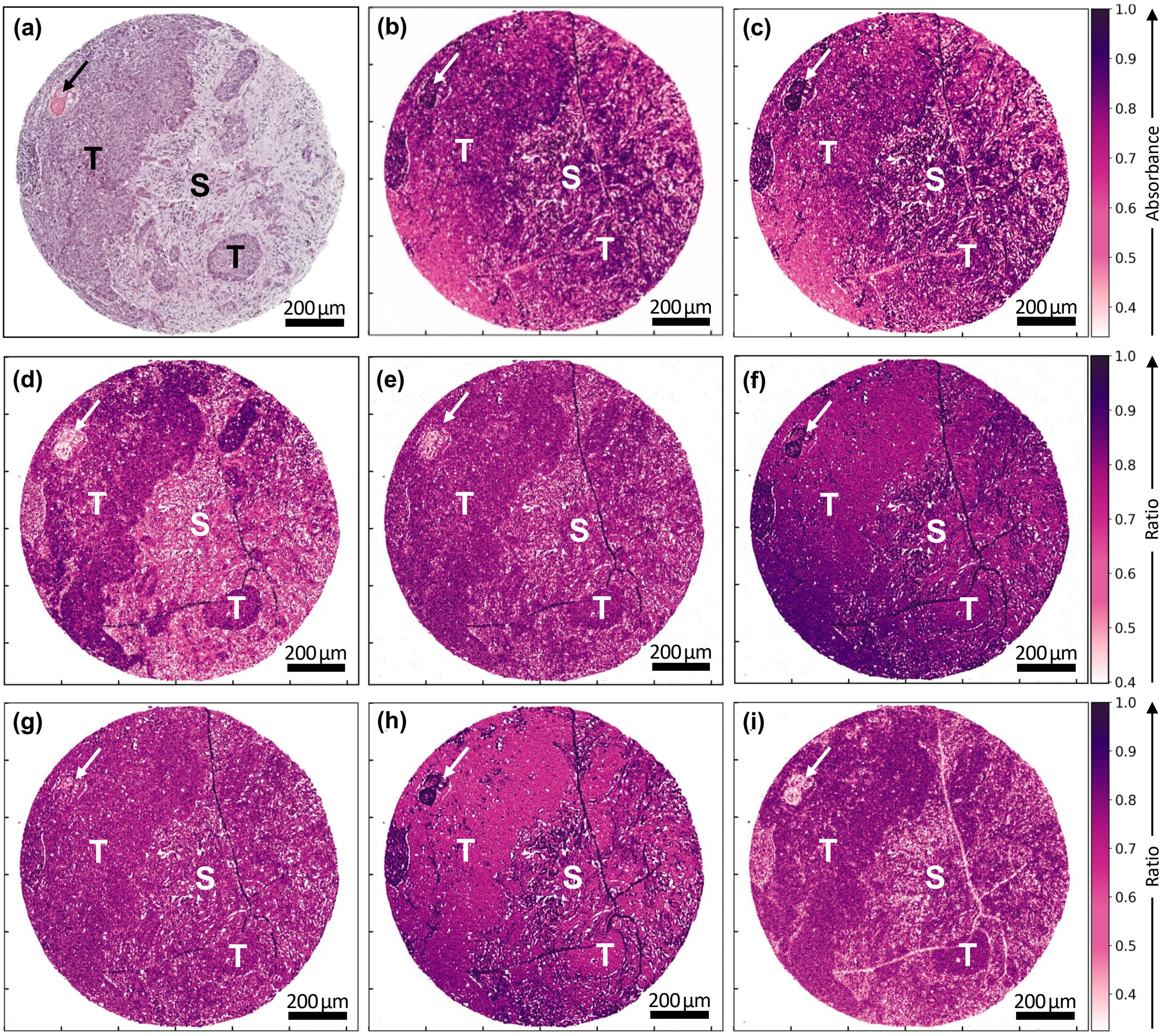

Figure S1: Comparison of H&E with O-PTIR images. (a) H&E image of a tissue core containing both OSCC and stroma with minimal lymphoid tissue; O-PTIR images at (b) 1252 cm^{-1}, (c) 1285 cm^{-1}; O-PTIR ratio images at (d) 1252 cm^{-1}/1285 cm^{-1}, (e) 1252 cm^{-1}/1540 cm^{-1}, (f) 1252 cm^{-1}/1660 cm^{-1}, (g) 1285 cm^{-1}/1540 cm^{-1} (h) 1285 cm^{-1}/1660 cm^{-1} and (i) 1660 cm^{-1}/1540 cm^{-1}. Key to tissue labels: stroma (S); OSCC tumour (T); keratin pearl (arrow).
